# Relative biocompatibility of micro-hybrid and nano-hybrid light-activated composite resins

**DOI:** 10.15171/joddd.2017.001

**Published:** 2017-03-15

**Authors:** Abiodun Olabisi Arigbede, Bukola Folasade Adeyemi, Omowumi Femi-Akinlosotu

**Affiliations:** ^1^Department of Restorative Dentistry, Faculty of Dentistry, University of Port Harcourt, Port Harcourt, Nigeria; ^2^Department of Oral Pathology, Faculty of Dentistry, College of Medicine, University of Ibadan, Ibadan, Oyo State, Nigeria; ^3^Department of Anatomy, Faculty of Basic Medical Sciences, College of Medicine, University of Ibadan, Ibadan, Oyo State, Nigeria

**Keywords:** Biocompatibility, composite resins, implantation

## Abstract

***Background.*** In vitro studies have revealed a direct association between resin content and cytotoxicity of composite resins; however, implantation studies in this regard are sparse. This study investigates the relationship between filler content of composite resins and biocompatibility.

***Methods.*** This research employed twelve 180‒200-gr male Wistar rats, 1 nano-hybrid (Prime-Dent Inc.) and 1 micro-hybrid (Medental Inc.) composite resins containing 74% and 80‒90% filler content, respectively. The samples were assessed on the 2nd, 14th and 90th day of implantation. Four rats were allocated to each day in this experimental study. A section of 1.5mm long cured nano-hybrid and micro-hybrid materials were implanted into the right and left upper and lower limbs of the rats, respectively. Eight samples were generated on each day of observation. Inflammation was graded according to the criteria suggested by Orstavik and Major. Pearson’s chi-squared test was employed to determine the relationship between the tissue responses of the two materials. Statistical significance was set at P < 0.05.

***Results***. The average grade of inflammation for the nano-hybrid on the 2nd day of implantation was 3.3. The micro-hybrid resin had a score of 3.0 for cellular inflammation. On the 14th day, the micro-hybrid resin also exhibited a lower average grade for cellular inflammation. On the 90th day, the micro-hybrid resin had a higher grade of inflammation (0.9) compared to 0.3 recorded for nano-hybrid. The composite resins with higher filler content elicited a significantly lower grade of inflammation irrespective of the duration (χ=20.000, df=8, P=0.010) while the composite resins with lower filler content elicited a significantly lower inflammatory response on the 90th day (χ=4.000, df=1, P=0.046).

***Conclusion.*** The composite resins with higher filler content generally elicited significantly lower grades of inflammation, and the composite resins with lower filler content exhibited significantly lower inflammatory response on the 90th day of implantation.

## Introduction

The introduction of composite-based resin technology to tooth restoration has been described as one of the most significant contributions to clinical dentistry in the last century.^1^ Composite resins are versatile tooth-colored restorative materials, and indeed the most widely used material for restoration of anterior teeth.^1,2^ Many years ago, amalgam was the most popular direct filling material for premolars and molars. This is because it could be easily placed, it has excellent mechanical properties, it is resistant to wear, and marginal leakage is not significant.^1,2^However, the use of the material has declined in the recent past while that of composite resins is on the rise even in stress-bearing areas like the molar region because of better aesthetics and biocompatibility.^3^ Major components of composite resins are the resin matrix and the inorganic fillers.^2^Incorporation of fillers into the resin matrix component improves the mechanical properties of this restorative material significantly.^2^ Composite resins use three types of fillers, including ground quartz, colloidal silica and glasses or ceramic containing heavy metals.^2^ Based on the size of filler particles, composite resins can be classified into macro-filled (conventional) resins, microfilled resins, hybrid resins, micro-hybrid resins, and nanofilled.^4^ Nano-composite resins may be categorized into three groups: true nano-filled resins, nano-hybrids and Ormocers (organic-modified ceramics).^4^Composite resins are also classifiedbased on their matrix components,^5^ including those containing conventional matrix where the chemical system is pure methacrylate, e.g. hybrid composite resin and nano composite resin; inorganic matrix where the chemical system is inorganic polycondensate (Ormocers); acid modified methacrylate where the chemical system is polar groups (Compomers); and those containing ring opening epoxide where the chemical system is cationic polymerisation.^5^ Recently, bulk-fill materials (BFMs) which are viscous and flowable have been marketed.^6^

Most composite resins utilize monomers that are aromatic or aliphatic diacrylates. Of these, bisphenol A glycidyl methacrylate (BisGMA) is probably the most extensively used, but urethane dimethacrylate is also frequently employed.^2^Polymerized composite resins have been found to leach residual monomers of undesirable biocompatibility.^7-9^ Methacrylate-based composite resins are believed to release uncured monomers into the tissues, which have been associated with hypersensitivity, cytotoxicity, genotoxicity, estrogencity and immune system alterations.^10^ Reports from in vitro studies^11,12^ revealed a direct association between resin content and cytotoxicity, but implantation studies in this regard are sparse. It is necessary to investigate this parameter in animal studies because the results obtained from different techniques were found to be inconsistent.^13^This study was therefore designed to investigate the relationship between filler content and inflammatory tissue reaction of nano-hybrid and micro-hybrid composites resins.

## Methods

This experimental study was conducted in the Departments of Anatomy and Oral Pathology of our College of Medicine and approval for the research was granted by the Ethical Review Committee on Experimental Animals of our University. One nano-hybrid light cured composite resin (Prime-Dent, Prime-Dent manufacturing, Inc, USA) and one micro-hybrid light cured composite resin (Medental, Medental International, Inc) were selected based on filler content and size (Table 1). Twelve healthy male Wistar rats weighing 180‒200gr were used in this experiment and the days of observation were 2nd, 14th and 90th days of implantation. Four rats were allocated to each day of observation. The implant materials were inserted into the subcutaneous tissues of the rat.^14-16^

**Table 1:** Composition of the resin composites employed

**Material**	**Content**	**% by weight** **(volume)**
**Prime-Dent (Nano-hybrid)** **Prime-Dent manufacturing, Inc, USA** **Lot No. BQJ12N**	Bis-GMA Filler(Average article size=0.70 μm)	(not stated) 74 (58.89)
**Medental (Micro-hybrid)** **Medental International, Inc** **Lot No. 20100914**	Silsne treated ceramic Bis GMA (BIS Phenol free) Triethyleneglycol (TEGDMA)	80-90 1-10 1-10

**Figure 1.**
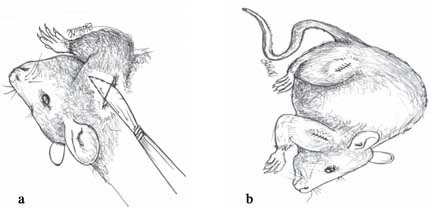
Blunt dissection (a); sutured implant site (b).

The resins were prepared according to the manufacturers’ recommendations and then cut into 1.-mm-long sections. The animals were sedated using intraperitoneal injection of ketamine/xylazine (90/10 mg/kg). Blunt forceps was used to lift the dorsal skin of the lateral sides of the upper and lower limbs of the animals; then a 1cm long incision was made along the length of the limbs. Furthermore, dissection of the subcutaneous tissue was carried out with a piece of thin-edged surgical scissors down to where the test materials were positioned (Figure 1a). Prime-Dent restorative material was implanted into the right limbs, while Medental restorative material was implanted into the left limbs. The incisions were then closed using a silk suture material Figure 1b) and the animals were closely observed.

On the 2nd, 14thand 90th days of implantation, respectively, the rats were again anaesthetized and the implanted materials were removed surgically along with the tissues in the immediate vicinity of the implant. The excised tissues were placed in appropriately labeled specimen bottles containing 10% neutral formalin solution. The tissues were processed as appropriate until they were converted into hematoxylin and eosin (H and E) stained slides, ready to be analyzed. All the slides were examined and rated by an experienced oral pathologist who was blinded to the dental material used and the period of investigation to avoid bias. For each of the 4 rats allocated to each day of observation, 2 different samples (from the upper and lower limbs) were obtained for each material under investigation yielding a total number of 8 samples.

Grading of tissue reaction

Tissue reactions to the implants were graded according to the criteria suggested by Orstavik and Major as follows:^14,17^

No inflammation: Tissues appeared normal. No inflammatory cells

Mild inflammation: Few inflammatory cells

Moderate inflammation: Increased reaction zone and more inflammatory cell infiltrate

Severe inflammation: Increased reaction zone and more intense inflammatory cell infiltrate

Extreme inflammation: Dense infiltration by inflammatory cells

The average grade of inflammation elicited by each material per day of observation was calculated as follows:

Ʃ [grade of inflammation (0-4) × f] /n (n=8)

To further assess the inflammatory tissue response, vascular congestion was graded according to the criteria employed by Marković et al.^18^The average grade of vascular congestion was also calculated as stated above.

### Data management

SPSS 16.0 was used to generate summary statistics. Pearson’s chi-squared test was used to assess the relationship between the tissue responses to each of the test materials. Statistical significance was set at P < 0.05.

## Results

**Figure 2.**
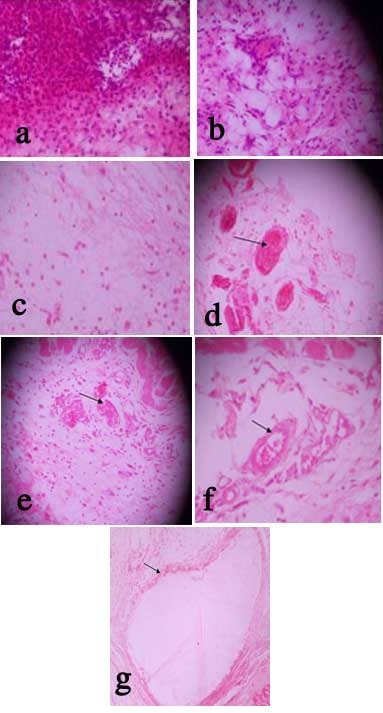
Photomicrographs showing dense inflammatory cell infiltration graded as ‘Extreme inflammation’ (×100) (a); moderate inflammatory cell infiltration graded as ‘Moderate inflammation’ (×100) (b); sparse inflammatory cell infiltration graded as ‘Mild inflammation’ (×100) (c); congested blood vessels (arrow) graded as ‘Severe congestion’ (×40) (d); vessels with moderate amount of red blood cells graded as ‘Moderate congestion’ (×100) (e); vessels that have very few red blood cells; fibrous band formed (f);and fibrous band formed around the sample (×100) associated with little or no inflammation on day 90, arrow pointing the fibrous capsule (g).

The distributions of the grades of inflammatory responses and vascular congestion are presented in Tables 2 and 3, respectively. On the second day of implantation, 50.0% of the sites implanted with Prime-Dent nano-hybrid composite resin exhibited extreme inflammation (characterized by infiltration of acute inflammatory cells) and 25.0% exhibited severe inflammation. The average grade of inflammation was 3.3. The majority (62.5%) of the sites of implantation exhibited mild vascular congestion, while only 12.5% exhibited moderate vascular congestion. The average grade of vascular congestion was 1.9. Similarly, 50.0% of the sites of implantation of Medental micro-hybrid exhibited features of extreme inflammation and 25.0% showed features of severe inflammatory cell infiltration. Unlike Prime-Dent, the remaining sites showed mild cellular inflammation. The average grade of cellular inflammation was 3.0. The majority (75.0%) of the sites of implantation had mild vascular congestion, while 25.0% displayed severe vascular congestion. The average grade of vascular congestion was 2.3. Samples of inflammatory response and vascular congestion recorded are presented in Figures. 2a-g.

**Table 2:** Distribution of grades of inflammatory response to implanted composite resins in relation to day of implantation(N=8)

**Material**	**No of Days**	**Grade of inflammation**					
		**Extreme/4** **f(%)**	**Severe/3** **f(%)**	**Moderate/2** **f(%)**	**Mild/2** **f(%)**	**None/0** **f(%)**	**Average** **f(%)**
**Prime=Dent**	2	4(50.0%)	2(25.0%)	2(25.0%)	-	-	3.3
	14	-	1(12.5%)	2(25.0%)	3(37.5%)	2(25.0%)	1.3
	90	-	-	-	2(25.0%)	6(75.0%)	0.3
**Medental**	2	4(50.0%)	2(25.0%)	-	2(25.0%)	-	3.0
	14	-	-	2(25.0%)	4(50.0%)	2(25.0%)	1.0
	90	-	-	-	7(87.5%)	1(12.5%)	0.9

**Table 3:** Distribution of grades of Vascular congestion in relation to day of implantation(N=8)

**Material**	**No of Days**	**Grade of inflammation**					
		**Extreme/4** **f(%)**	**Severe/3** **f(%)**	**Moderate/2** **f(%)**	**Mild/2** **f(%)**	**None/0** **f(%)**	**Average** **f(%)**
**Prime=Dent**	2	**-**	1(12.5%)	5(62.5%)	2(25.0%)	**-**	1.9
	14	**-**	**-**	1(12.5%)	**-**	7(87.5%)	0.3
	90	**-**	**-**	-	1(12.5%)	7(87.5%)	0.1
	2	**-**	2(25.0%)	6(75.0%)	**-**	**-**	2.3
	14	**-**	**-**	-	1(12.5%)	7(87.5%)	0.1
	90	**-**	**-**	-	1(12.5%)	7(87.5%)	0.1

Furthermore, on the 14^th^day of implantation of Prime-Dent nano-hybrid composite, severe inflammation, characterized by chronic inflammatory cellular infiltration comprising macrophages, lymphocytes and a few plasma cells, were seen in 12.5% of the sites of implantation, while 25.0% elicited mild inflammation. The average grade of cellular inflammation was 1.3, while the average grade of vascular congestion was 0.3. For Medental micro-hybrid composite resin, on the other hand, 50.0% of the implant sites showed mild cellular inflammation, while 25.0% elicited moderate inflammation. The average grade of cellular inflammation was 1.0, and 87.5% of the sites of implantation did not exhibit evidence of vascular congestion (Tables 2and3).

Prime-Dent material showed mild chronic cellular infiltration on the 90th day of implantation in 28.6% of the sites and the average grade of cellular inflammation was 0.3. In addition, 12.5% of the implant sites showed minimal vascular congestion. For Medental micro-hybrid, however, 87.5% of the sites exhibited mild chronic cellular reaction on the 90th day of implantation. The average grade of cellular inflammation was 0.9 and minimal congestion was seen in 12.5% of the cases (Tables2and3).

Pearson’s chi-squared test showed a significant difference in cellular response to the two implants, with Medental having a lower response compared to Primedent (χ=20.000, df=8, P = 0.010) irrespective of the duration of the implantation. On day 2, (χ=4.000, df=3, P = 0.261) and fourteen days of implantation (χ=1.143, df=3, P = 0.767) no significant difference was recorded in the cellular response to the test materials. On day 90, the cellular response to Priemdent was significantly less than that to Medental (χ=4.000, df=1, P = 0.046). Concerning vascular response, a significant difference was not noted in response to the materials for days two (χ=2.424, df=2, P = 0.298), fourteen (χ=2.000, df2, P=0.368) and ninety (χ=0.000, df=1, P = 1.000), respectively.

## Discussion

The chemicals that are released by composite resins and by-products of biodegradation of the material may elicit local and systemic adverse reactions. One of the clinically relevant tests for assessing local tissue reaction to a foreign material is implantation study.^19-21^ The local adverse reaction comprises pulpal and mucosal effects, which may include irritation, inflammation, epithelial proliferation and oral lichenoid reactions. The present study showed that Medental micro-hybrid composite resins elicited a slightly lower cellular inflammatory response on the 2nd and 14th days, but the vascular congestion was slightly higher on the 2nd day of implantation. As reported in culture experiments,^11,12^ the difference in the cellular inflammatory responses may be related to the higher filler content in the Medental product. Ergun et al^11^discovered that the composite resins that had the highest rate of survival among the five tested composite resins was the one with the highest weight (87%) of filler content. Srivastava et al^12^also reported that flowable composite resins with higher resin content showed maximum toxicity compared to nano composite resin and compomer.

However, the cellular inflammatory response of Prime-Dent resins was much lower on the 90th day of inflammation. The reason for this was not clear at the moment; it may be due to the difference in the resin matrix composition and reactions between the two materials. Prime-Dent nano-hybrid contains BisGMA, while Medental micro-hybrid contains BisGMA (phenol free) and TEGDMA. BisGMA and TEGDMA have been reported as the most toxic resin based components, respectively.^8,9,23^It is noteworthy that the BisGMA in Medental composite resin is free of phenol. Bisphenol A and BisGMA have both been confirmed as estrogenic chemicals.^9,22^

The 2nd, 14th and 90th days of observation were employed in the present study because they were suitable for observing the initial responses and to see whether chronic inflammation is ongoing or it has resolved.^14,23^The attendant inflammation that followed tissue trauma that occurred during surgical implantation of materials is not distinguishable from that elicited by the implanted materials under investigation.^18,23^ Since the two test materials were implanted in the same experimental animal using the same technique, the overall results remained valid even without control. This study was limited by the small sample size employed. Further studies that would employ large sample sizes are recommended. In addition, it is important to study this subject further because it has been reported^24^ that the quantity and configuration of implanted samples influence tissue reactions. The difference in tissue responses may be due to the difference in percentage composition of the resin content and the types of resins in the materials.

## Conclusion

The two composite resins displayed acceptable level of biocompatibility. Composite resins with higher filler content elicited significantly lower grades of inflammation on the whole, but composite resins with lower filler content elicited significantly lower inflammatory response on the 90th day of implantation.

## Acknowledgments

The authors thank all our friends and members of staff of our departments, who encouraged us to complete the study.

## Authors’ contributions

The concept and the design of the study were developed by AOA. The implantation, care of the animals and the harvest, were carried out by OFA. The slides were prepared and interpreted by BFA. Data entry and statistical analyses, too, were carried out by BFA. The manuscript was written by AOA. The proof reading was carried out by BFA and OFA. All the authors participated in the literature review.

## Funding

The study was sponsored by the authors. The authors received no funding from any other individual or institution.

## Competing interests

The authors declare no competing interests with regards to the authorship and/or publication of this article.

## Ethics approval

The ethical approval for the research was granted by the Ethical Review Committee on Experimental Animals of University of Ibadan.
